# Academic Clinical Trials Submitted to an Institutional Ethics Committee at a Tertiary Care Center: A Retrospective Analysis

**DOI:** 10.7759/cureus.53476

**Published:** 2024-02-02

**Authors:** Yashashri C Shetty, Yashoda R Aithal, Janhavi Katkar, Manali Naik

**Affiliations:** 1 Pharmacology and Therapeutics, Seth Gordhandas Sunderdas (GS) Medical College and King Edward Memorial (KEM) Hospital, Mumbai, IND; 2 Institutional Ethics Committee, Seth Gordhandas Sunderdas (GS) Medical College and King Edward Memorial (KEM) Hospital, Mumbai, IND

**Keywords:** academic clinical trials, new drugs and clinical trials rules 2019, clinical trials, drugs controller general of india, central licensing authority, investigator-initiated studies

## Abstract

Background

The role of academia in clinical research has given rise to the concept of academic clinical trials (ACTs), which are vital in generating evidence. Through the implementation of the New Drugs and Clinical Trials Rules-2019 (NDCTR-2019) rules, the Institutional Ethics Committee (IEC) has obtained a quasi-regulatory role. The study aims to assess the challenges the IEC faced when processing, approving, and monitoring ACTs. The other objectives included the number of ACTs submitted to the IEC, as well as administrative, scientific, and ethical issues stated by the IEC and the Drug Controller General of India (DCGI) authorities. We also aimed to provide some insight into the type of decision made by IEC and DCGI - the delay or inconsistency between the queries.

Methods

This retrospective study was conducted in the IEC of a tertiary care hospital, Mumbai, Maharashtra, India. A comprehensive search of the IEC database was carried out by the study team, and only those protocols of ACTs submitted to IEC between January 2015 and December 2021 were included. The studies submitted between January 2015 and February 2019, i.e., before the release of NDCTR-2019, were classified as the "Before" category. All subsequently submitted protocols were grouped together as the "After" group. Descriptive statistics were used to represent the data, while comparison between the two timeframes were made using the Mann-Whitney U test with a level of significance at 5%.

Results

This six-year study showed that merely 1.4% (34/2400) trials fulfilled the criteria of an ACT. An increase in the ACT protocol submission was noted in the “After” group (20 vs. 14). Most ACTs were drug trials, with 67.6% (23/34) trials conducted majorly in the Department of Anesthesiology. There was a statistical increase in time query reply by the principal investigator to IEC and the time between submission and approval in the “After” group (p<0.05). IEC sent out 94 administrative, 565 scientific, and 216 ethical queries. On IEC monitoring, protocol deviations were noted; nonetheless, no ACTs reported protocol deviations or serious adverse events.

Conclusions

Since the implementation of NDCTR-2019, IEC has taken on a quasi-regulatory function, and there has been an increase in the caliber of IEC monitoring and adherence to ethical norms.

## Introduction

Three considerations should ideally guide advancements in healthcare decisions: the clinician's expertise, the patient's preferences and values, and the best external evidence, such as findings from clinical trials (CTs) [1]. Thus, in addition to assessing the safety as well as efficacy of a novel drug or device, CTs can provide evidence-based support for patient care [2].

The idea that pharmaceutical industry trials solely create novel drugs for financial and commercial gain is certainly widespread. Consequently, industry-sponsored research and studies are frequently criticized and seen to be possibly biased and profit-oriented [3]. Pharmaceutical sponsors plan and oversee trials that are carried out by investigators who are in charge of the daily operations [4]. The gap between the needs of clinical practice and disease burden is sometimes not answered by pharmaceutical CT.

Investigator-initiated studies (IIS) or academic clinical trials (ACTs), are carried out by academicians and are required to address several important clinical questions [5]. Academicians who are actively engaged in patient care, teaching, administration, and research execute a CT. They play a dual role in an ACT, being both the sponsor and the investigator, and one can personally oversee adherence to regulations [6]. ACT involves a wide range of studies including trials for new drugs, and real-world prospective or retrospective studies that aid clinicians in repurposing drugs and carrying out research that are pertinent to their practice, hence generalizable to the population where the study was conducted. With the evidence generated from ACTs, it is possible to build hospital-, state-, and country-specific health guidelines and policies. The other benefits include conversion of drug label from off-label to labelled drugs. Additionally, academicians' careers advance when an IIS is conducted since the researchers make every attempt to publish the findings as soon as possible [3,7,8]. While ACTs emphasize the effectiveness of the intervention, CTs frequently demonstrate the efficacy of the intervention under study. ACTs offer potential benefits by broadening the horizon of product knowledge, encompassing safety. It is believed that non-industry sources of information are always given greater importance [9].

Thus, all of these issues were brought to light by the New Drugs and Clinical Trials Rules-2019 (NDCTR-2019), which defined the ACT as “clinical trial of a drug already approved for a certain claim and initiated by any investigator, academic or research institution for a new indication or new route of administration or new dose or new dosage form, where the results of such a trial are intended to be used only for academic or research purposes and not for seeking approval of the Central Licensing Authority (CLA) or regulatory authority of any country for marketing or commercial purpose” [10].

As announced by the regulatory authority, ACT will require Institutional Ethics Committee (IEC) approval, as stated in GSR 313 (E), dated March 16, 2016, and the generated data will not be submitted to the regulator. The IEC can wait an extra 30 days requesting the regulator for such research. The IEC would approve the research if there was no response from the Drug Controller General of India (DCGI) within that period [11]. However, the NDCTR-2019 stated that no permission for conducting an ACT shall be required for any drug from the CLA when trials are conducted on permitted drug formulation is intended solely for academic research purposes for a new indication or new route of administration or new dose or new dosage form; and has been initiated after prior approval by the IEC for CT. The concerned IEC shall notify the CLA in writing indicating its views within 30 working days of the receipt of the application in the event of a potential overlap between the ACT and CT or a doubt regarding the nature of the study [10]. The regulator has now granted IEC a quasi-regulatory position for authorizing these ACTs, which calls for IEC to be sufficiently qualified to make the right choices and have the necessary control.

However, in ACTs, the investigators encounter a variety of hurdles during the research, including funding, constant surveillance, training of the study team, lack of expertise in statistical procedures, data management, and medical writing [7]. The IEC, in addition to the investigator, will play a crucial role in assuring adherence to ethical standards, the quality of the data collected, and the safety of the intervention through activities including monitoring, auditing, and the Data Safety Monitoring Board. To keep their members up to date, they are warranted to call for timely, suitable training [8].

The ethics committee at Seth Gordhandas Sunderdas Medical College and King Edward Memorial Hospital accepts IIS protocols from a variety of specialties and operates around-the-clock. Due to the dearth of data, we decided to conduct a study to better understand the challenges the IEC faced when processing, approving, and monitoring ACTs. We aimed to assess the number of ACTs submitted to IEC, as well as administrative, scientific, and ethical issues stated by IEC and the DCGI authorities. We also wanted to provide some insight into the type of decision made by IEC and DCGI - the delay and inconsistency between the queries. Additionally, we contrasted the timetables followed by IEC and DCGI before and after the introduction of NDCTR-2019.

## Materials and methods

This study employed a retrospective, observational approach to assess ACT protocols that were submitted to the IEC. An expedited approval was granted by the IEC of Seth Gordhandas Sunderdas Medical College and the King Edward Memorial Hospital, Mumbai (IEC(II)OUT/131/2020). The research team followed the standard operating procedures (SOPs) for document recovery, and a thorough analysis was completed in the institute's Office of IEC. Only those studies deemed as ACTs were retrieved from the IEC database and included in the current study. A confidentiality agreement with IEC was signed by the study team to protect the privacy of the participants and investigators of such ACTs. The documents including the demographic details of ACTs (number of ACTs submitted, department of conduct of ACTs, area of research), functioning of IEC (time taken by IEC to review and the response sent to the investigator), review process of IEC and DCGI (the nature of queries sent to the investigator - administrative, scientific, or ethical), and the outcome of trials (decision, number of serious adverse events (SAEs) that occurred, compensation paid by the investigator, monitoring undertaken during the trial conduct, number and nature of protocol deviations (PDs), number of trial termination and completions) were all carefully analyzed by the study team between January 2015 and December 2021. The studies submitted between January 2015 and February 2019, i.e., before the release of NDCTR-2019, were classified as the "Before" category. All subsequently submitted protocols were grouped together as the "After" group.

Statistical analysis

Microsoft Excel 365 (Microsoft Corp., Redmond, WA) was used to record the information received for each ACT, and descriptive analysis of the demographic data was performed. Frequency and percentages were used to describe categorical data, whereas mean ± standard deviation or median (interquartile range) were used to express continuous data. The Mann-Whitney U test was used for comparisons at 5% level of significance.

## Results

Over the period of six years, 2,400 studies were submitted to the IEC for approval. Only 34 studies, 34/2,400 (1.41%), met the criteria for ACTs after a thorough review of every protocol submitted to IEC. We noted an increase in the number of protocols being submitted to IEC in the "After" group (20 versus 14 studies in the "Before" group). Furthermore, it was observed that drug trials made up around 67.6% (23/34). This was followed by device trials, 11.76% (4/34), trials on diagnostic procedures, 5.88% (2/34), and trials on surgical techniques, 5.88% (2/34). Three studies with the label "others" included one multi-ingredient trial and two single-drug herbal trials.

Drug trials included a change in drug dose, 39.1% (9/23), a change in drug indication, 39.1% (9/23), a change in drug formulation, 4.3% (1/23), and the use of some drugs in fixed-drug combinations, 17.4% (4/23). In addition, 19 out of the 34 studies were approved by IEC, of which only three trials were registered under the Clinical Trial Registry India (CTRI) website. Around 17.6% (6/34) studies were sponsored by extramural grants; however, only 5.9% (2/34) studies had an intramural funding. It was found that 19 of the 34 trials submitted to IEC stated that they would make use of departmental funding, whereas 7/34 did not mention the source or the process of application for sponsorship.

Table 1 summarizes the departments involved in ACTs in the institution. Most of the research, accounting for 38.1%, was conducted by the Department of Anesthesiology with the objective of improving hemodynamics during anesthesia by either lowering the dosage of the anesthetic drug or addition of an analgesic for improved pain management or overall perioperative management.

**Table 1:** Distribution of ACTs among specialty branches. ACT, academic clinical trial

	Number (N=21)	Percentage %
Specialty branches		
Anesthesia	8	38.09
Dermatology	4	19.04
Biochemistry	2	9.52
Orthopedics	2	9.52
Pediatrics	2	4.76
Otorhinolaryngology	1	4.76
Pharmacology	1	4.76
Obstetrics and gynecology	1	4.76
Superspecialty branches		
Plastic surgery	2	15.38
Nephrology	2	15.38
Endocrinology	2	15.38
Gastrointestinal medicine	2	15.38
Surgical gastroenterology	1	7.69
Neurosurgery	1	7.69
Urology	1	7.69
Pediatric surgery	1	7.69
Clinical pharmacology	1	7.69

**Figure 1:**
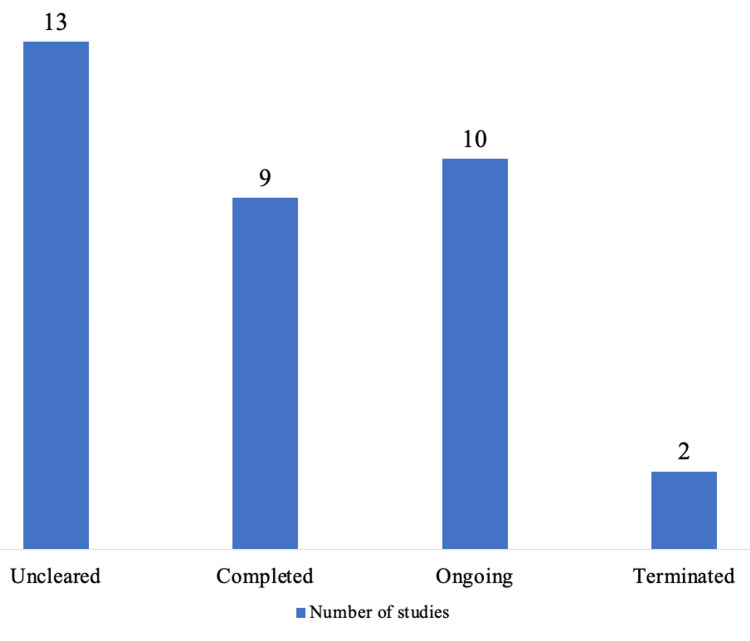
Status of ACTs submitted to IEC. ACT, academic clinical trial, IEC, Institutional Ethics Committee

Figure 1 depicts the status of ACTs submitted to IEC.

Two trials conducted in the "Before" and "After" periods, however, were terminated. Only 3/20 (15%) trials in the "After" group and 6/14 (42.8%) trials in the "Before" group were successfully completed. At the time of the study team's analysis, 13 studies were uncleared, one in the "Before" group and 12 in the "After" group. One study in the "Before" category was uncleared because DCGI suggested that the protocol be resubmitted as a device study rather than a drug trial. In the "After" group, 12 studies were deemed uncleared. The primary causes were failing to sign a Memorandum of Understanding (MoU) (2/12) and missing insurance policy for the trial (3/12). Ten trials were still ongoing in the "After" group.

The functioning of IEC was analyzed employing several parameters; the details are tabulated in Table 2.

**Table 2:** Functioning of IEC **Signifies p<0.05 using the Mann-Whitney test DCGI, Drugs Controller General of India; IEC, Institutional Ethics Committee; NA, not applicable; SD, standard deviation

Turnaround time	Before, mean ± SD (days), N=14	After mean ± SD (days), N=20	p-Value
Submission to the first query letter (IEC)	41.71±10.76	50.85 ± 5.86	0.345
Query reply by the principal investigator to IEC	81.15±10.76	180.84± 5.86	0.015**
Submission to approval from IEC	226.83±80	420.5±45	0.015**
No. of letters from IEC	2.45± 0.934	2.28 ±1.109	0.245
Submission to the first query letter (DCGI)	57.33 ± 19.82	NA	NA

In the "Before" group, 2.6 ± 1 query letters were sent by IEC to the principal investigator (PI), compared to 2.4± 0.92 letters in the "After" group. With a p>0.05, there was no significant difference between the two groups.

**Figure 2:**
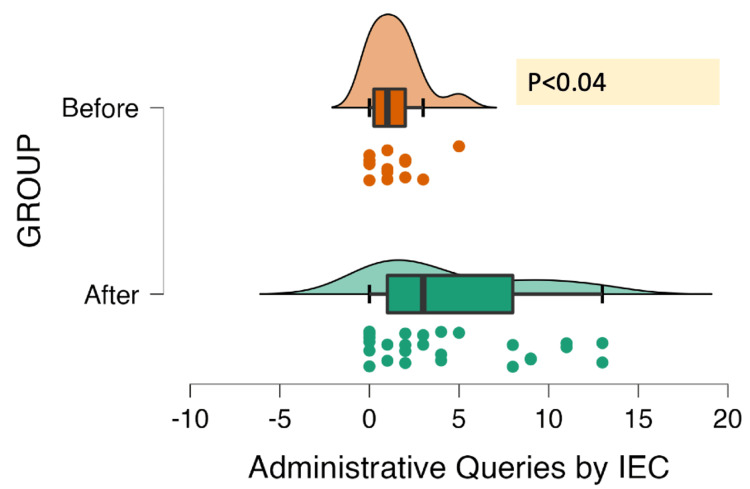
Distribution of administrative queries (N=34 ACTs; total=94 queries) ACT, academic clinical trial; IEC, Institutional Ethics Committee

**Figure 3:**
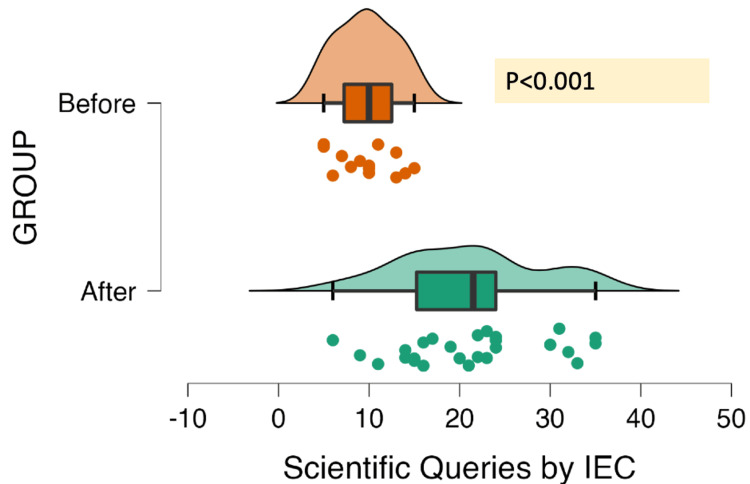
Distribution of scientific queries (N=34 ACTs; total= 565 queries) ACT, academic clinical trial; IEC, Institutional Ethics Committee

**Figure 4:**
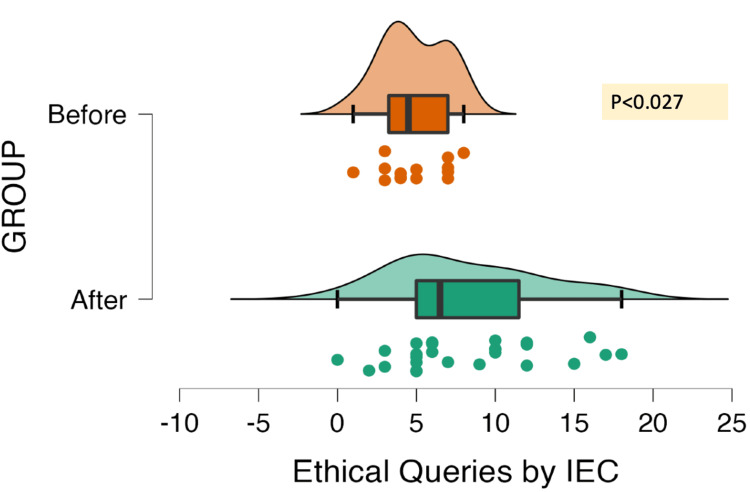
Distribution of ethical queries (N=34 ACTs; total= 216 queries) ACT, academic clinical trial; IEC, Institutional Ethics Committee

The IEC/DCGI's queries were essentially categorized as being of the administrative type, scientific type, or ethical type. Figures 2-4 show the kind of queries that were submitted to the PI across 34 different studies. There were 94 administrative, 565 scientific, and 216 ethical queries sent in all. A statistically significant difference between the "Before" and "After" groups was found using the Mann-Whitney test, considering the type of query.

A total of 9 and 15 administrative queries were sent out to the PI in the "Before" and "After" groups, respectively. Common queries raised to the PI concerned the documentation of the study team, collaborative study without seeking permission, package insert issues, the absence of an MoU and an investigator undertaking, the inaccessibility of updated Good Clinical Practice training and Maharashtra Medical Council certificates, and the lack of DCGI permission. In addition to the aforementioned queries, information on insurance coverage and funding was raised in the "After" group.

IEC sent out approximately seven "Before" and 15 "After" queries regarding the scientific shortcomings, which included a deficient review of the literature, unclear objectives, endpoints, recruitment strategy, a method of randomization and blinding, the standard of care used, sample size calculation method, statistical analysis used, storage of samples, and specifics of laboratory processing. Clarification of risk-benefit analysis, failure to provide protocol-specific investigations or treatments, compensation, and changes to the informed consent document (ICD) and/or procedure were the key ethical issues in 10 "Before" and 12 "After" trials. The queries from DCGI were concerned toward the details of the investigational product (source, manufacturer details, license), toxicological and pharmacological data, details of trial stakeholders, and details of IEC approval (details of the deliberations and minutes of meeting). Additionally, DCGI gave IEC instructions to submit a proposal for evaluation, and a telephonic call was received from DCGI to present two project protocols before the subject expert committee and provide clarifications to DCGI. Nevertheless, following the DCGI remarks, these studies, which had previously received IEC approval, were placed on hold.

When the 34 ACT conducted in our institution were analyzed for their outcomes, none of the studies in either timeframe revealed any SAEs or PDs. IEC monitored the conduct of one trial in each of the two groups and found issues in ICD and case record form (CRF). Concerns raised in the ICD were related to the inclusion of a legally authorized representative when not necessary or the omission of an impartial witness' signature in the case of an illiterate participant. Either the PI did not sign the CRFs in the research or used an incorrect version of CRF for the trial.

## Discussion

The clinical research strength of a nation is based on its academic studies. These studies are essential for producing pertinent, objective data that will be used to implement new treatments and help health authorities make more informed decisions. Several articles have been published emphasizing the challenges and perspectives of investigators in the conduct of an ACT. Although the concept of ACTs initially came up in 2015, changes were made to the regulations with the introduction of NDCTR-2019. The set of comparisons was done in the "Before" and "After" periods because in the "Before" period the IEC's operation under DCGI supervision and in the "After" period its functioning as a regulator were primarily examined in this study. But this is the first study of its sort that highlights the difficulties the IEC had in reviewing, authorizing, and overseeing the ACT.

In the current research, a meagre 1.41% of the total studies submitted to the IEC met the criteria for ACTs after a thorough review, highlighting a dearth of the ACTs. This suggests a lack of experience in research methodologies, as well as a lack of proficiency in statistical knowledge, data management, and medical writing. There is also a lack of motivation to conduct innovative research along with a lack of knowledge regarding regulatory guidance. The setting up of clinical trial units (CTUs), which can govern the entire research arena of the institution, ranging from training, delegating investigators with appropriate CTs, creating facilities for research, to increasing collaboration across clinical and paraclinical departments, can enhance the planning and implementation of the ACT. Bhide et al. undertook research to assess investigators’ awareness and understanding of ACTs among tertiary institutions in Mumbai. The results of this study showed that individuals were less aware of the laws and regulations that are particular to ACTs. Furthermore, the investigators made references to matters concerning participant recruiting, funding, logistics, and Ethics Committee (EC)-related difficulties in conducting ACTs [12]. Teaching academicians about the essentials of research methodology, manuscript writing, training in SOPs of IEC, and revised legislation and norms from regulatory bodies are some potential solutions to the aforementioned problems [7].

Compared to the "Before" group, the "After" group submitted a notably higher number of ACTs to the IEC (20 vs. 14 studies). This increase in the number might be attributed to the change in the NDCTR-2019 rules. This mentions that if an investigational product is intended only for academic research purposes for a new indication, new route of administration, new dose, or new dosage form, it has received prior approval from the EC, and the results of the ACT are not used for the promotional activities, then no permission for conducting an academic CT shall be required for any drug from the CLA. Therefore, the ease of the ACT's norms in NDCTR-2019 and IEC’s role as a regulator encourages academicians to plan and carry out many such ACTs [13].

According to the current study, the majority of submitted trials were drug trials that sought to alter drug dose, indications, formulations, or the use of certain medications in fixed drug combinations, among other things. Because there were fewer device and diagnostic procedure usages in our institution, the trials pertaining to the same were lesser in numbers. Through these ACTs, academicians discover new uses, adapt dosage schedules, apply the drug or technology to new patient populations, and offer a clinical evaluation of benefits and hazards. Therefore, the results of ACTs may expand the use of off-label drugs and be beneficial, especially when all other recognized therapeutic options have been exhausted, as is occasionally the case with rare conditions [14]. In the current study, most research was carried out at the Department of Anesthesiology, followed by Department of Dermatology. For the comfort, safety, and hemodynamic stability of their patients, an anesthesiologist administers several medications, including sedatives, analgesics, anesthetics, and adjuvant drugs. Depending on the experience and preference of the physician, medications may occasionally be used in doses, methods, or indications that differ regulatory drug approval [15]. Furthermore, a number of dermatological illnesses (such as xanthelasma palpebrarum and melasma) are so uncommon or not curable (such as vitiligo) that no approved options for treatment exist, indicating an unmet need. Off-label drug use frequently yields valuable scientific information that influences or even enhances subsequent therapeutic conceptions [16]. The research conducted by Jobanputra et al. and Manjesh et al. revealed a significant incidence of off-label and unlicensed medication use among pediatric patients. It is also believed that because of their financial interests, pharmaceutical sponsors are unwilling to fund research on such regimens. However, in order to convert from off-label to on-label use, it is imperative that numerous ACTs be conducted and produce insightful data that will direct future medical professionals. Thus, ACTs demonstrate a cutting-edge advantage for the generation of evidence under such circumstances. Therefore, it is essential that an investigator and DCGI discuss the trial's outcomes [17,18].

A total of 13 studies, one in the "Before" group and 12 in the "After" group, were deemed "uncleared” because of administrative problems, such as those involving agreements, insurance, and so on. Following the formation of NDCTR-2019, the IEC's regulatory function has been effective in approving the trial. Though the 19 studies were approved by IEC, the study highlighted a lower number of registration of trials (just 3 ACTs) on the CTRI website. We believed that registering through CTRI would reinforce and have an impact on the final reporting of CTs as well as their design and ethical conduct. The investigators may not have voluntarily reported ACTs on the CTRI website due to a lack of time, knowledge, or motivation. Furthermore, fewer ACTs were registered in CTRI because of the perception that the registration process was cumbersome and WHO 20 data set field needs to completed with additional CTRI-specific information. By contrast, a decadal perspective by Rao et al. shows that pharmaceutical-funded CTs have more trials documented on CTRI [19]. The discrepancy in the data may be explained by the availability of trained and experienced staff, as well as by time and necessary tasks to be completed prior to the initial patient recruitment in the pharmaceutical CTs. Furthermore, in the present setting, the majority of biomedical journals need a full CTRI registration at the time the paper is published [20,21]. This is supported by the findings of our investigation where the status of publications of the nine completed ACTs is not known. Among the options put out to address these issues include training in manuscript writing, mandatory submission of CTRI registration by the IEC, and familiarization with and updates to the CTRI website. A further suggestion about the assessment of the publishing status of all completed CTs by the IEC and the provision of academic awards and credits to investigator by the institute is also recommended.

This research showed that around 17% ACTs were carried with extramural grants, especially those sponsored by pharmaceutical sectors. Government and nonprofit funding for ACT has declined during the past years, while pharmaceutical industrial investment has surged globally. There are two sides to these pharmaceutical-aided ACTs. Academicians will benefit in terms of research funds from the pharmaceutical sponsors, and they will also advance professionally because of taking on the position of PI. However, persistent shadowing of the pharmaceutical sector may have a negative impact as the trials get dangerously influenced by their commercial interests and might take precedence above both the ethical obligation to patients and the scientific intent [22,23]. Furthermore, these sectors gain a backdoor access into the pharmaceutical market through ACTs, bypassing the need to invest time, money, manpower, or ethical responsibility. The resistance to conducting an ACT among academicians can be significantly decreased by offering an effective infrastructure and sufficient funds through internal or external sponsorship via government agencies, non-governmental agencies institutional funding, or corporate social responsibilities of the pharmaceutical industry [24].

The present study’s results demonstrated an increasing trend in the turnaround times for submitting the initial IEC query letter, the query reply, the submission for IEC approval, and the total number of inquiry letters that the IEC sent out. Since the NDCTR-2019 regulations were established, the IEC had statistically increased the number of queries sent to the investigator, including those of an administrative, scientific, and ethical character. This is because the IEC oversees, monitors, and audits an ACT in order to fulfil its quasi-regulatory function. This implies that regular training on institutional SOPs, protocol writing, grant/insurance/MoU application procedures, and any updated ACT rules and regulations should be provided to the site employees/investigators. It is important to motivate academicians to set aside time for research and training in research methodology. In the end, this would result in fewer queries, which would speed up research approval. Additionally, a loss of motivation and interest in the research is caused by delays in approval or an increase in the number of queries. Another reason could be that the current retrospective analysis covered the period between January 2015 and December 2021. As the tenure of membership in EC was for three years, half of the members were replaced after each term. The NDCTR guidelines stipulated that 50% of the members of the EC must be non-institutional and the rest must be from the institution. Non-institutional members ought to comprise a greater proportion of the membership if the total number of members is odd. The NDCTR-2019 implementation featured various new requirements for EC members, including deadlines and training. This might be the reason for faster timelines followed by the EC members.

The study reported no serious adverse events (SAEs) or PDs. However, deviations were found during monitoring in ICD and CRF, hinting at the efficiency of IEC. For all ACTs, or at least those considered high-risk interventions or those involving vulnerable populations, such monitoring visits must be regularly carried out by IEC. According to an audit carried out at our institute's IEC by Jalgaonkar et al., a total of 447 PDs were filed in the IEC, with 323 major PDs and 124 minor PDs. It was noted that 281/323 major deviations were from pharmaceutical sectors, while a negligible 36/323 deviations were documented by ACTs. [25]. A similar study by Tripathi et al. reported reduced reporting of SAE from IIS, demonstrating a poor compliance with SAE reporting among the PIs, which is consistent with current research [26]. A higher frequency of PDs in the pharmaceutical sector is because of the regular monitoring and a dedicated group of clinical scientists who work continuously to establish protocols and carry out studies. However, in ACTs, these deviations and failure to report SAEs might compromise the study outcomes.

The study is constrained by its single-centered design and retrospective study methodology. Since this study was carried out at a public hospital, we are unable to determine with certainty how EC operates in independent and private ECs. Identifying ACTs in research EC settings was a challenge. Therefore, we speculate that few of the studies may have been missed from the analysis, leading to analysis bias. Given that none of the completed ACTs got published, concerns about publication bias may have been raised.

From the study findings, the authors recommend setting up a CTU within the organization, which can facilitate the trial's efficient operation. These CTUs can provide additional support to the investigators in the trial's design, documentation, and execution. Additionally, provision of training in IEC SOPs, and regulations and guidelines pertaining to CT conduct and research methodology periodically are recommended. It is of utmost importance to assign equal importance to academic research as to patient care and medical education by the institution. Generation of funds by institutional grants and provision of time to academicians for focused research are further recommended. Further prospective multi-centric studies assessing the functions of EC should be carried out for all the ACTs conducted in the public and private sectors.

## Conclusions

The implementation of NDCTR-2019 has improved quality of the adherence to ethical standards, the quality of the data collected, and the safety of the intervention due to quasi-the regulatory role of IEC. This audit demonstrated the negligible number of ACTs conducted at our institute, with maximum being drug trials carried out at the Department of Anesthesiology. Efficiency of IEC's operations was noted after NDCTR-2019 implementation, as evidenced by increased queries sent out and a turnaround time.
